# Ab Initio Studies of Mechanical, Dynamical, and Thermodynamic Properties of Fe-Pt Alloys

**DOI:** 10.3390/ma17153879

**Published:** 2024-08-05

**Authors:** Ndanduleni Lesley Lethole, Patrick Mukumba

**Affiliations:** Department of Physics, University of Fort Hare, Private Bag X1314, Alice 5700, South Africa; pmukumba@ufh.ac.za

**Keywords:** Fe-Pt alloys, elastic constants, Debye temperature, phonon dispersion curves, stability, mechanical stability, thermodynamic properties

## Abstract

The density functional theory (DFT) framework in the generalized gradient approximation (GGA) was employed to study the mechanical, dynamical, and thermodynamic properties of the ordered bimetallic Fe-Pt alloys with stoichiometric structures Fe_3_Pt, FePt, and FePt_3_. These alloys exhibit remarkable magnetic properties, high coercivity, excellent chemical stability, high magnetization, and corrosion resistance, making them potential candidates for application in high-density magnetic storage devices, magnetic recording media, and spintronic devices. The calculations of elastic constants showed that all the considered Fe-Pt alloys satisfy the Born necessary conditions for mechanical stability. Calculations on macroscopic elastic moduli showed that Fe-Pt alloys are ductile and characterized by greater resistance to deformation and volume change under external shearing forces. Furthermore, Fe-Pt alloys exhibit significant anisotropy due to variations in elastic constants and deviation of the universal anisotropy index value from zero. The equiatomic FePt showed dynamical stability, while the others showed softening of soft modes along high symmetry lines in the Brillouin zone. Moreover, from the phonon densities of states, we observed that Fe atomic vibrations are dominant at higher frequencies in Fe-rich compositions, while Pt vibrations are prevalent in Pt-rich.

## 1. Introduction

Ferromagnetic Fe-Pt intermetallic alloys are a significant group of solid-state materials, exhibiting intriguing physical and chemical properties that are inherently dependent on their specific chemical compositions. These materials find applications across various industries, including catalysis for chemical reactions, ultrahigh-density magnetic data storage such as hard disk drives and magnetic sensors, spintronic devices, and high-temperature mechanics due to their large uniaxial magnetocrystalline anisotropies (MCA) and high coercivity, amongst other desirable magnetic properties [1,2,3,4]. The large MCA presents the potential of decreasing the magnetic particle dimensions to a few nanometers of grain sizes without degrading the thermal stability of the magnetization axis direction. Crystallographically, they stabilize in two Strukturbericht symbols and three space groups: L1_0_ (body-centered tetragonal P4/mmm-FePt) and L1_2_ (face-centered cubic $Pm\bar{3}m$-Fe_3_Pt, $Pm\bar{3}m$-FePt_3_ and body-centered tetragonal I4/mmm-Fe_3_Pt). The L1_0_ phase features an alternating arrangement of Fe and Pt atoms which can undergo a disorder–order phase transition from face-centered cubic (fcc) to tetragonal at temperatures of approximately above 650 °C [5]. Müller et al. employed both analytic bond-order potential and lattice-based Monte Carlo simulations and reported that the observed disorder–order transition temperature decreases with particle grain size [6,7]. Furthermore, Yu et al. reported that the ferromagnetic orientation and dynamical stability break down above the threshold pressure of 96.7 GPa as a result of undergoing spontaneous magnetization [8]. The transformation has significant implications for understanding the dynamical properties of Fe-Pt alloys, as it influences the material’s magnetic behavior band mechanical response. Furthermore, in L1_0_ bimetallic compounds, the difference in atomic size between the two constituent atoms is less than 15%. Thus, the orientation of magnetization and magnitude of the local magnetic moment and stability are significantly influenced by the concentrations and atomic sizes of Fe and Pt [9]. These dissimilarities in crystal lattice arrangement and chemical compositions significantly impact the lattice dynamics of these alloys. The face-centered Fe-rich $Pm\bar{3}m$-Fe_3_Pt has a similar intermetallic arrangement as the Cu_3_Ag-ordered crystal structure. Temperature-based X-ray diffraction and transmission electron microscopy studies demonstrated that the $Pm\bar{3}m$-Fe_3_Pt alloy fully crystallizes at annealing temperatures above 800 ℃ [10,11]. The grain size and lattice parameter increase until this temperature and remain constant thereafter, signifying that crystallization is fully converged and lattice has reached stability. Moreover, the Mössbauer spectrometry and magnetic characterization results showed that the structure conforms to a ferromagnetic state at temperatures below 120 K and paramagnetic state at 300 K and above. The L1_2_ cubic Pt-rich $Pm\bar{3}m$-FePt_3_ is an intermediate alloy that occurs below 300 ℃ during the formation of the L1_0_ FePt [4]. Further structural arrangements were discussed in detail in our previous communication [3]. Moreover, recently revised phase diagram studies have revealed the existence of other novel ordered FePt_2_ and Fe_2_Pt alloys [12]. Yu et al. have since performed computational simulations on these alloys to investigate their various thermodynamic, mechanical, and dynamical stabilities [13,14]. For the FePt_2_ composition, they reported on five different space groups, namely: I4/mmm, P4/nmm, Immm, Cmcm, and P63/mmc, which were predicted to be thermodynamically and mechanically stable, with the I4/mmm being the most thermodynamically stable [13]. Furthermore, four space groups of Fe_2_Pt, namely: Cmcm, Immm, I4/mmm, and $P\bar{3}m1$ were also reported to be low-energy ferromagnetic stable structures [14].

Phonon dispersion spectra measurements of Fe-Pt alloys to determine their dynamical properties have previously been reported using both experimental and density functional theory (DFT)-based first-principles studies. Pierron-Bohnes et al. measured the vibrational modes of the conventional unit cell of the ordered L1_0_ P4/mmm-FePt structure at 300 K using the inelastic neutron scattering method [15]. They observed that this alloy is thermodynamically stable since the dispersion relations curves displayed only positive modes of vibrations. Earlier, Noda et al. also investigated and compared the dispersion spectra between the ferromagnetic $Pm\bar{3}m$-Fe_3_Pt and paramagnetic $Pm\bar{3}m$-FePt_3_ cubic L1_2_ alloys using the inelastic neutron scattering technique at room temperature [16]. They observed that the acoustic modes for both alloys are similar, particularly in the lower-energy region, mainly because their lattice constants differ by only 3%. It has been also determined that the dispersion curves in Fe_3_Pt and FePt_3_ are influenced by the force constants associated with their respective nearest neighbors, namely Fe-Fe and Pt-Pt; thus, the nearest neighbor pairs have dominant roles in lattice dynamics. Sternik et al. corroborated the experimental measurements by conducting first-principles computations on the phonon dispersion curves of the 2 × 2 × 2 primitive supercells of P4/mmm-FePt, $Pm\bar{3}m$-Fe_3_Pt, and $Pm\bar{3}m$-FePt_3_ alloys using the direct method as embedded in the PHONON code [17]. They reported that the P4/mmm-FePt and $Pm\bar{3}m$-FePt_3_ alloys show no imaginary modes along high symmetry directions of the Brillouin zone, while there are imaginary frequencies for the transversal acoustic mode on the $Pm\bar{3}m$-Fe_3_Pt alloy. Furthermore, their investigation into the impact of magnetic ordering on the dispersion relations of $Pm\bar{3}m$-FePt_3_ revealed that the frequencies of the antiferromagnetic orientation slightly surpass those of the ferromagnetic orientation. Nevertheless, the dispersion curves exhibit a remarkable similarity.

Investigation of mechanical properties such as elasticity, hardness, shear resistance, ductility, and anisotropy is a crucial prerequisite in solid-state materials before their design and development. DFT calculations of elastic constants have been performed for various binary and ternary alloys and were reported to reveal valuable insights at both ambient and elevated conditions. Phasha et al. investigated the structural and mechanical properties of the ordered Mg-Li binary alloys to predict their phase stability [18]. Their findings revealed that there is a correlation between the heats of formation and elastic constants in terms of predicting stability. Phases with negative heats of formation tend to satisfy the Born necessary stability conditions and have a positive tetragonal shear modulus $C^{\prime }$. Recent DFT studies on various alloys and other compounds have also corroborated these pioneering conclusions. Botha et al. investigated the relationship between the mechanical, dynamical, and thermodynamic properties of Pt-Pd alloys by calculating their elastic constants, phonon dispersion spectra, and heats of formation, respectively [19]. Their results indicated that energetically favorable, i.e., with negative heats of formation, compositions satisfy Born stability conditions (mechanical stability) and depict only positive modes of vibrations in the phonon dispersion spectra (dynamical stability). Similar studies were performed on Co-alloyed MnPt alloys, revealing that energetically favorable compositions are mechanically stable [20,21]. It was further revealed that the Debye temperature of CsGaSb_2_ calculated from the single-crystal elastic constants yields approximately a similar value to the one calculated from the phonon density of states, confirming a robust link between mechanical and dynamical properties. Moreover, the polycrystalline bulk modulus calculated from the elastic constants exhibited exceptional alignment with values derived from alternative equations of state [21]. DFT calculations on the elastic constants of Fe-Pt alloys (P4/mmm-FePt, $Pm\bar{3}m$-Fe_3_Pt, and $Pm\bar{3}m$-FePt_3_) have also been previously performed [11]. It was reported that $Pm\bar{3}m$-Fe_3_Pt is mechanically unstable due to negative $C^{\prime }$ and $C_{44}$ values, while others satisfied the Born stability conditions. On the contrary, earlier results revealed mechanical stability [7,22].

Despite the significant experimental and computational advances made in these alloys, there remains a notable gap regarding the origins of lattice dynamics and elastic behavior for optimizing their functionality and ensuring their long-term stability. Moreover, little work has been completed on the I4/mmm-Fe_3_Pt alloy, prompting the need to understand and compare the mechanical, dynamical, and thermodynamical properties across the entire spectrum of existing Fe-Pt alloys. Thus, the current communication will put special emphasis on this alloy. These properties are critical in designing and developing new composition-dependent magnetic materials. Additionally, understanding these properties provides valuable insights in predicting the stability of these materials when subjected to industrial conditions. Building from our previous communication [3] where the structural, magnetic, and electronic properties of P4/mmm-FePt, I4/mmm-Fe_3_Pt, $Pm\bar{3}m$-Fe_3_Pt, and $Pm\bar{3}m$-FePt_3_ were reported, the current study presents density functional theory (DFT)-based first-principles computations to establish a corroboration between the mechanical, dynamical, and thermodynamic properties of the same alloys at ambient conditions. Elemental contributions of Fe and Pt atoms on the phonon dispersion spectra are also determined by the partial phonon density of states. Moreover, this work is an extrapolation of the existing theoretical and inelastic neutron scattering measurements of magnetic, mechanical and lattice dynamical properties of Fe-Pt alloys which have reported TA-mode softening on $Pm\bar{3}m$-Fe_3_Pt [11,17,23,24]. We found that the equiatomic P4/mmm-FePt alloy exhibits dynamical stability, as evidenced by only positive vibrations in the phonon dispersion spectrum. Moreover, the less-studied I4/mmm-Fe_3_Pt alloy shows comparable mechanical stability, ductility, anisotropy, and Debye temperature with the other alloys. Our findings contribute to the growing body of knowledge in the field of alloy research and provide a solid foundation for future investigations. By unravelling the complexities of these ordered alloys, we can unlock their full potential and harness their properties for technological advancements.

## 2. Computational Procedure

All calculations were performed using the Cambridge Serial Total Energy Package (CASTEP) [25] simulation code which is incorporated in with the Materials Studio 2020 version suite. CASTEP is a widely used code in the field of computational chemistry and materials science due to its ability to accurately predict the properties and behaviors of materials at the atomic level. Its key strength lies in its ability to perform first-principles calculations based on density functional theory, which accurately describes the electronic structure of materials without the need for empirical parameters, thus allowing the study of properties such as elastic constants, phonon spectra, and thermodynamic properties. However, some calculations are time-consuming and computationally expensive, limiting the size of systems that can be studied and the timescales that can be simulated. We carried out collinear spin polarization density functional theory (DFT) computations within the generalized gradient approximation (GGA) utilizing the Perdew, Burke, and Ernzerhof (PBE) functional [26]. All structures were initially relaxed by performing a full geometry optimization while maintaining a fixed basis quality and allowing cell volume to change. The Coulomb interactions between the valence electrons of iron (Fe: 3d^6^ 4s^2^) and platinum (Pt: 4f^14^ 5d^9^ 6s^1^) atoms, and their respective pseudo-ionic cores, were accurately modelled using on-the-fly generated (OTFG) ultrasoft pseudopotentials. This approach enables calculations to be conducted with reduced energy cut-offs and ensures that the generated potentials are steady across solid-state and pseudo-atom calculations. Furthermore, it enhances the accurateness and dependability of the results by utilizing the same exchange-correlation functional throughout. A customized energy cut-off of 350 eV was adequate to minimize the total energy of the bulk structure until the difference between two successive low-memory Broyden–Fletcher–Goldfarb–Shanno (BFGS) iterations was within 0.001 eV. The low-memory Broyden–Fletcher–Goldfarb–Shanno (LBFGS) [27] algorithm was preferred due to its ability to accelerate geometry optimization and accuracy for large systems [28]. To sample the wave functions, we employed the Monkhorst-Pack grid parameters of 9 × 9 × 7, 4 × 4 × 4, 6 × 6 × 6, and 8 × 8 × 8 for P4/mmm-FePt, I4/mmm-Fe_3_Pt, $Pm\bar{3}m$-FePt_3_, and $Pm\bar{3}m$-Fe_3_Pt, respectively. The calculations for all structures were conducted assuming the ferromagnetic (FM) arrangement of the local magnetic moments. Moreover, for the tetragonal P4/mmm-FePt and I4/mmm-Fe_3_Pt, we computed the primitive tetragonal cell as opposed to the conventional cell due to minimal computational resources. As a result, I4/mmm-Fe_3_Pt with lattice parameters a≠c was computed as I4/mmm-Fe_3_Pt with a=c. The phonon dispersion spectra along high symmetry lines and the accompanying phonon density of states were calculated via the finite displacement method which was proven to be highly effective for metallic systems [29,30]. The finite displacement technique relies on numerically differentiating forces acting on atoms, which are calculated for multiple unit cells with atomic displacements. Phonon density of states also serves as a requirement for the computation of thermodynamic properties, which permits assignment of temperature factors during analysis. Lastly, the monocrystalline elastic constants were calculated using the stress–strain approach with a maximum strain amplitude of 0.003. The additional calculation criteria for elastic constants involved sustaining total energy of convergence below $2.0\times 10^{-6}\;eV/atom$, making sure that the Hellman–Feynman force is maintained under 0.006 eV/Å, and keeping the ionic displacement within $2.0\times 10^{-4}\;Å$.

## 3. Results and Discussion

### 3.1. Mechanical Properties

Before determining the mechanical and dynamical properties of Fe-Pt alloys, structural relaxation was performed to obtain ground-state lattice parameters. To validate the approach utilized, the calculated structural lattice parameters were compared with the existing experimental data. The purpose of this analysis is to ensure the accuracy and reliability of our results. The current GGA-calculated lattice parameters exhibit an impressive agreement of over 98% with the previously reported data, thereby demonstrating the robustness of the approach employed.

Table 1 presents the calculated lattice constants, elastic constants, moduli, Pugh ratio, anisotropy factor, Poisson ratio, Vickers hardness, and Debye temperature for the Fe-Pt alloys. Calculation of elastic constants is crucial in characterizing the mechanical behavior of materials and response to externally induced stress. The Taylor expansion of the total energy of a strained system [31,32] (see Equation (1)) was used to calculate the elastic constants (C_ij_).

(1) $$U\left(V,\varepsilon \right)=U\left(V_{0},0\right)+V_{0}\left[\sum_{i}\tau_{i}\varepsilon_{i}\xi_{i}+\frac{1}{2}\sum_{ij}C_{ij}\varepsilon_{i}\xi_{i}\varepsilon_{j}\xi_{j}\right],$$

where $U\left(V_{0},0\right)$ is the energy of the unstrained system with equilibrium volume $V_{0}$; $\tau_{i}$ and $\xi_{i}$ are elements in the stress tensor and a factor taking care of the Voigt index, respectively. Cubic and tetragonal crystal systems contain three ($c_{11},c_{12},c_{44}$) and six ($c_{11},c_{33},c_{44},c_{66},\;c_{12}$,$c_{13}$) independent elastic constants, respectively. Except for $C_{12}$ in P4/mmm-FePt, the obtained monocrystalline elastic constants are in agreement with the previous semi-empirical Monte Carlo results obtained using the modified embedded atom method (MEAM) and angular dependent analytic bond-order potential (ABOP) formalism [6,22], further affirming the accuracy of the approach employed. Moreover, our $C_{12}$ value is in better agreement with the DFT value of 94 GPa reported by Zotov and Ludwig [11]. For cubic crystals ($Pm\bar{3}m$-Fe_3_Pt and $Pm\bar{3}m$-FePt_3_) to be deemed mechanically stable, the mandatory Born stability criterion in Equation (2) must be fulfilled [33,34].

(2) $$C_{11}+2C_{12}>0,\;{\;C}_{11}>\left|C_{12}\right|\;\;and\;\;C_{44}>0$$

The mechanical stability conditions for tetragonal systems (P4/mmm-FePt and I4/mmm-Fe_3_Pt) are delineated based on Equation (3) [35]. We note that the elastic constants for both the cubic and tetragonal systems are satisfied, demonstrating mechanical stability, which corroborates the thermodynamic stability reported previously [3]. Additionally, we have noted a positive $C^{\prime }$ value, which further confirms the mechanical stability of the systems.

(3) $$C_{11}-C_{12}>0;\;\;C_{11}+C_{33}-2C_{13}>0;\;\;\;C_{ii}>0\;\left(i=1,\;3,\;4\right);\;\;2C_{11}+C_{33}+2C_{12}+4C_{13}>0$$

The elastic constants of the tetragonal systems can further be analyzed by examining their similarities and discrepancies. Significant differences are observed between the calculated $C_{ij}$ values, suggesting that the tetragonal alloys are highly anisotropic. Particularly, $C_{11}$ and $C_{33}$ are significantly higher than $C_{44}$ and $C_{66}$, indicating greater resistance to unidirectional compression compared to shear. Furthermore, there exists a substantial difference between the following pairs of $C_{ij}$s, $C_{11}$ and $C_{33}$; $C_{44}$ and $C_{66}$; $C_{12}$ and $C_{13}$, which has an impact on the behavior of the material. $C_{11}$ represents the stiffness of the material in the direction perpendicular to the crystallographic planes, while $C_{33}$ represents the stiffness parallel to the crystallographic planes, namely [100]; [010]; and [001]. $C_{11}$ values are significantly higher than those of $C_{33}$, indicating greater resistance to compression in the a-axis in comparison to the c-axis or greater linear compressibility along the c-axis compared to the a-axis when the material is subjected to external forces. Thus, the interatomic chemical bonds within the (001) plane are more robust compared to the bonding along the crystal direction [001]. The relationship $C_{44}>C_{66}$, indicates that shear deformation is more prominent when a net stress is imposed along the [100] crystallographic direction of the (010) crystallographic plane, as opposed to when the same stresses are exerted in the [100] direction within the plane (001). This indicates that the alloys exhibit anisotropic behavior with greater resistance to shear deformation in one direction compared to another. Moreover, the significant difference between $C_{12}$ and $C_{13}$ demonstrates that when stress is imposed along the a-axis, the subsequent strain will be more pronounced along the a-axis than the c-axis. This further highlights the anisotropic response of these alloys under stress, as evidenced by the high $A^{U}$ values. When assessing the level of anisotropy, we have chosen to utilize the universal anisotropy index $A^{U}$ in Equation (4) as postulated by Ranganathan and Ostoja-Starzewski [36]. This index is particularly suitable as it considers both shear and bulk contributions, acknowledging the inherent anisotropy present in all single crystals. The magnitude of the deviation of $A^{U}$ from zero is the measure of the degree of anisotropy exhibited by a single crystal, with a value of zero indicating a locally isotropic crystal.

(4) $$A^{U}=5\frac{G^{V}}{G^{R}}+\frac{B^{V}}{B^{R}}-6$$

where $G^{V}$, $G^{R}$, $B^{V}$, and $B^{R}$ are the shear and bulk moduli Voigt and Reuss estimates, respectively. We observe that $A^{U}$ decreases with Fe content, thus $Pm\bar{3}m$-Fe_3_Pt and I4/mmm-Fe_3_Pt are highly anisotropic over P4/mmm-FePt and $Pm\bar{3}m$-FeP_3_, respectively.

The calculation of independent elastic constants allowed us to estimate the macroscopic elastic bulk (B), shear (G), and Young’s (E) moduli utilizing the Voigt [37] and Reuss [38] estimates. These estimates establish simple and linear relationships between the isotropic bulk and shear moduli of the polycrystalline material. Additionally, Hill demonstrated that the Voigt and Reuss expressions serve as upper and lower bounds, respectively, and proposed an arithmetic average modulus value [39]. The bulk modulus signifies the material’s ability to withstand compression under applied hydrostatic pressure, while the shear modulus indicates its resistance to deformation from external forces at a constant volume. Young’s modulus, on the other hand, measures the material’s stiffness by determining the ratio of vertical linear stress to linear strain.

(5) $$G_{V}=\left(\frac{C_{11}-C_{12}+3C_{44}}{5}\right);\;G_{R}=\left(\frac{C_{44}\left(C_{11}-C_{12}\right)}{4C_{44}+3\left(C_{11}-C_{12}\right)}\right);\;G_{H}=\left(\frac{G_{V}+G_{R}}{2}\right)$$

(6) $$B_{V}=\frac{1}{9}\left[2\left(C_{11}+C_{12}\right)+C_{33}+4C_{13}\right];B_{R}=\frac{C^{2}}{M};C^{2}=\left(C_{11}+C_{12}\right)C_{33}-2C_{13}^{1};\;\;B_{H}=\left(\frac{B_{V}+B_{R}}{2}\right)$$

(7) $$C^{\prime }=\left(\frac{C_{11}-C_{12}}{2}\right)$$

The current DFT calculations revealed that Fe-Pt alloys have intrinsic mechanical hardness, stiffness, and shear resistance, i.e., are highly resistant to changes from external mechanical stress due to relatively large magnitudes of B, G, and E. The shear modulus is less than the bulk in all the alloys, suggesting they exhibit greater resistance to volumetric change than shear deformation, and that the shear modulus is the parameter limiting stability. $Pm\bar{3}m$-FePt_3_ possesses the highest bulk modulus value, suggesting the greatest mechanical hardness and compression resistance over P4/mmm-FePt, $Pm\bar{3}m$-Fe_3_Pt, and I4/mmm-Fe_3_Pt, respectively. This is consistent with a previous DFT study on Pt/Pd alloys, which showed that the bulk modulus is higher in Pt-rich compositions [19]. The $Pm\bar{3}m$-Fe_3_Pt system possesses the lowest shear modulus value of 46.379 GPa, indicating the greatest susceptibility to shear deformation compared to I4/mmm-Fe_3_Pt, $Pm\bar{3}m$-FePt_3_, and P4/mmm-FePt, respectively. The Young’s modulus suggests that P4/mmm-FePt adopts highest stiffness over, $Pm\bar{3}m$-FePt_3_, $Pm\bar{3}m$-Fe_3_Pt, and I4/mmm-Fe_3_Pt, respectively.

**Figure 1:**
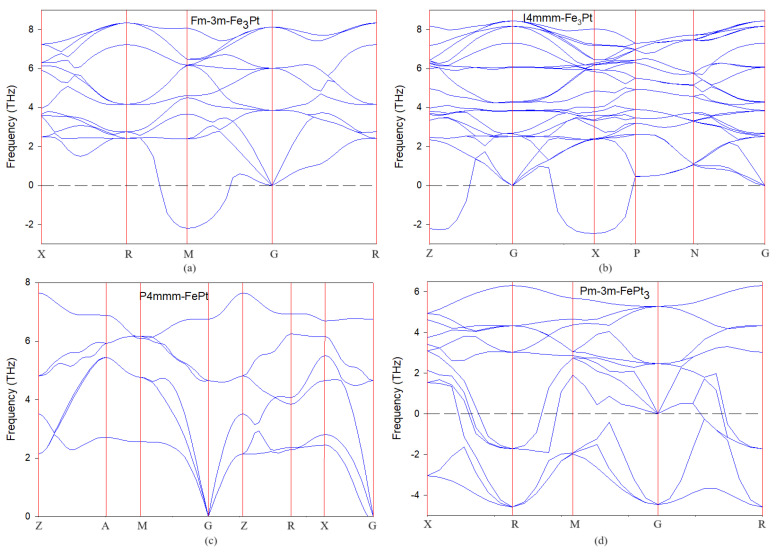
Phonon dispersion curves for (**a**) $Fm\bar{3}m$-Fe_3_Pt, (**b**) 4mmm-Fe_3_Pt, (**c**) P4/mmm-FePt, and (**d**) $Pm\bar{3}m$-FePt_3_ alloys.

To assess ductility and brittles, we computed the Pugh shear to the bulk modulus (*K*) [40] and Poisson (υ) [41] ration of solid-state materials. Pugh postulated that a material is considered ductile if *K* is less than the critical value 0.5 and brittle when greater than 0.5. We noticed that K values are less than 0.5 for all Fe-Pt alloys, demonstrating ductility. Ductile materials exhibit greater resistance to thermal shock and are easier to machine. Moreover, we noted that the Fe-rich $Pm\bar{3}m$-Fe_3_Pt and I4/mmm-Fe_3_Pt systems possess lower K values, due to their low shear modulus. The Poisson’s ratio associated with volume changes in directions perpendicular to the applied tension during deformation typically ranges from −1 to 0.5. An indication of ductility is present when the value exceeds 0.26, otherwise the material is considered brittle. The Poisson ratio for these alloys is greater than 0.26, further confirming ductility. Interestingly, the less-studied I4/mmm-Fe_3_Pt shows excellent ductility over $Pm\bar{3}m$-FePt_3_ and P4/mmm-FePt, respectively. Furthermore, the Poisson ratio was used to analyze bonding type in Fe-Pt alloys. Crystals are considered covalent if υ is approximately 0.1, ionic if υ=0.25, and metallic if υ>0.33 [42,43]. We note that υ values are greater than 0.33 for $Pm\bar{3}m$-Fe_3_Pt, I4/mmm-Fe_3_Pt, and $Pm\bar{3}m$-FePt_3_, implying metallic bonding. On the other hand, P4/mmm-FePt shows a ratio less than 0.33 but greater than 0.25, indicating half-metallic behavior. This is in good agreement with the density of states predictions reported in our previous communication [3]. To gain insight into the thermal properties of Fe-Pt alloys, we determined the Debye temperature ($\theta_{D}$). This property represents the point at which the atoms within a solid material cease to vibrate independently, but instead begin to move in a synchronized manner. It signifies the highest normal mode of vibration and serves to establish a correlation between elastic constants and phonon dispersions, specific heat, thermal expansion, and thermal conductivity. Equation (8) was utilized in the computation of the Debye temperature [44].

(8) $$\theta_{D}=\frac{\hbar \nu }{k_{B}}{\left(6\pi^{2}n\right)}^{1/3},$$

where n, ν, and $k_{B}$ are the atom concentration, phase velocity, and Boltzmann constant, respectively. Our DFT calculations show relatively huge magnitudes of Debye temperature (~300 K), indicating high vibrational modes in the phonon dispersion spectra (see Figure 1) and consequently high thermal conductivity. Laureti et al. employed the Correlated Debye Model (CDMT) to measure the Debye temperature of P4/mmm-FePt and reported $\theta_{D}=340\;K$ [4], which is consistent with our results (330.79 K). Materials with higher Debye temperatures also have stiffer and more rigid lattices, while those with lower Debye temperatures exhibit softer and more flexible structures [45,46]. To estimate the Vickers hardness of Fe-Pt alloys, we employed the microscopic bond resistance model proposed by Tian et al. [47] as shown in Equation (9).

(9) $$H_{V}=0.92k^{1.137}G^{0.708},$$

where k is the G/B ratio and G is the shear modulus. Our findings show that the metallic Fe-Pt alloys relatively show low hardness. In principle, this is expected for ductile materials with low (<0.5) G/B values, as proposed by Chen et al. [48].

### 3.2. Phonon Dispersion Curves

The phonon dispersion relations along high symmetry lines in the Brillouin zone were calculated to determine the lattice dynamic stability of Fe-Pt alloys and are presented in Figure 1. Phonon dispersion frequencies are calculated as the analysis of forces associated with a systematic set of atomic displacements from equilibrium positions in a crystal system. Materials are considered stable if there are no negative frequencies (soft modes) along high symmetry lines. We observe that the ordered L1_0_ P4/mmm-FePt displays only positive phonon vibrational modes along the high symmetry directions, indicating that all the eigenvalues are real and the alloy is dynamically stable. This finding aligns well with previous theoretical and experimental research [15,17,49]. The other compositions, in particular $Pm\bar{3}m$-FePt_3_, show imaginary frequencies of vibration along high symmetry lines in the spectrum, reflecting deviation dynamical instability and possible structural deformation which results in lowering of the symmetry [50]. The anomalous behavior shows that the frequencies of vibration decrease with the increasing wave vector as opposed to increasing. This may be attributed to the presence of complex interactions between the Fe and Pt atoms in the lattice, leading to the formation of localized vibrations that propagate through the material in a non-traditional manner. Dynamical instability is not pronounced for $Pm\bar{3}m$-Fe_3_Pt and I4/mmm-Fe_3_Pt since the negative vibrations are not along the center of the Brillouin zone G.

The modes of vibration for $Pm\bar{3}m$-Fe_3_Pt and I4/mmm-Fe_3_Pt as depicted in Figure 1a,b share a related frequency range and similarities along G, which can be attributed to their identical chemical composition and stoichiometry. Moreover, we note that the separation between the acoustic and optical modes is not enough to create a gap in the dispersion relations of all the alloys, suggesting seamless continuous energy transfer between the phonon modes. The disparities in mass among the alloys are evident through the observation that the frequencies of vibrations (optical modes) are higher in Fe-rich (with smaller mass) alloys and lower in Pt-rich (with larger mass) alloys.

### 3.3. Phonon Density of States

**Figure 2:**
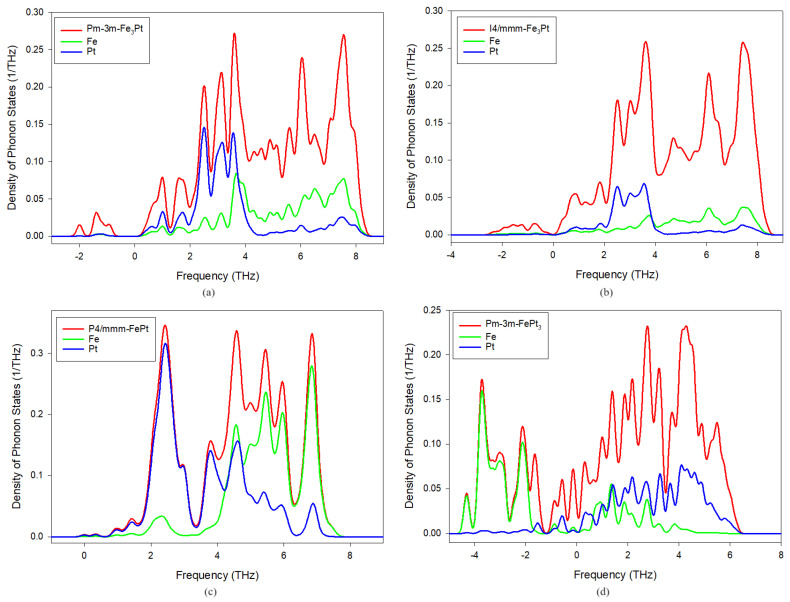
Calculated phonon density of states (**a**) $Pm\bar{3}m$-Fe_3_Pt, (**b**) I4/mmm-Fe_3_Pt, (**c**) P4/mmm-FePt, and (**d**) $Pm\bar{3}m$-FePt_3_ alloys.

To analyze the distinctive elemental vibrational contributions, we conducted calculations of the phonon density of states (DOS) as illustrated in Figure 2. The spectra can be categorized into two frequency regions: one where Fe vibrations predominate and another where Pt vibrations are more prominent. In Fe-rich compositions, Fe vibrations are prevalent at higher frequencies, whereas Pt-rich compositions exhibit dominance of Pt vibrations. Additionally, a significant level of anisotropy in lattice dynamics is apparent in all structures, particularly in P4/mmm-FePt (Figure 2c). The lowest frequency band (1.9–3.6 THz) comprises vibrations of the heavier platinum atoms, while frequencies above 4 THz are attributed to Fe vibrations. These findings align well with similar DFT calculations reported in previous studies [17,50]. In the Fe-rich $Pm\bar{3}m$-Fe_3_Pt and I4/mm-Fe_3_Pt compositions, Pt movement prevails up to 4 THz with minimal contribution from Fe, indicating that heavier Pt movement is responsible for the presence of imaginary soft modes leading to dynamical instability. Conversely, in the $Pm\bar{3}m$-FePt_3_ composition, Fe vibrations dominate in the −5 to 2 THz range, while Pt is more prominent in the 4 to 6 THz region. Therefore, the negative frequencies observed in the phonon dispersion curves of this alloy stem from the movement of multiple Fe atoms. Moreover, it should be noted that the elemental partial DOSs of Fe and Pt were sampled for only one atom each. Hence, there are significant differences between the sum (red) and atomic plots (blue and green) in the non-equiatomic compositions since other atoms are not included.

### 3.4. Thermodynamic Properties

Thermodynamic (temperature-dependent) quantities have been evaluated from the phonon density of states (DOS) in the temperature range of 0 to 1000 K based on the relations developed by Baroni et al. as listed in Table 2 [51]. All quantities were calculated at ground state, that is, geometry optimization was fully converged and all the phonon eigenfrequencies were real and non-negative.

Enthalpy, free energy, and entropy are fundamental thermodynamic properties that govern the behavior of solid-state materials. In the context of solid-state materials, enthalpy plays a crucial role in understanding phase transitions, such as melting and crystallization. For example, the enthalpy change associated with the melting of a solid reflects the energy required to overcome intermolecular forces and disrupt the crystalline structure, leading to a transition from a solid to a liquid state. Free energy is a key parameter in predicting the stability and equilibrium of different crystal structures. By comparing the free energy of different crystal phases, we can determine the phase that is thermodynamically favored under specific conditions. Entropy is closely related to the organization of atoms and molecules in a crystal lattice. Higher entropy is associated with increased disorder and greater freedom of movement for particles, whereas lower entropy corresponds to a more ordered and structured arrangement which is associated with favorable thermal conductivity and resistance to thermal expansion and deformation.

**Figure 3:**
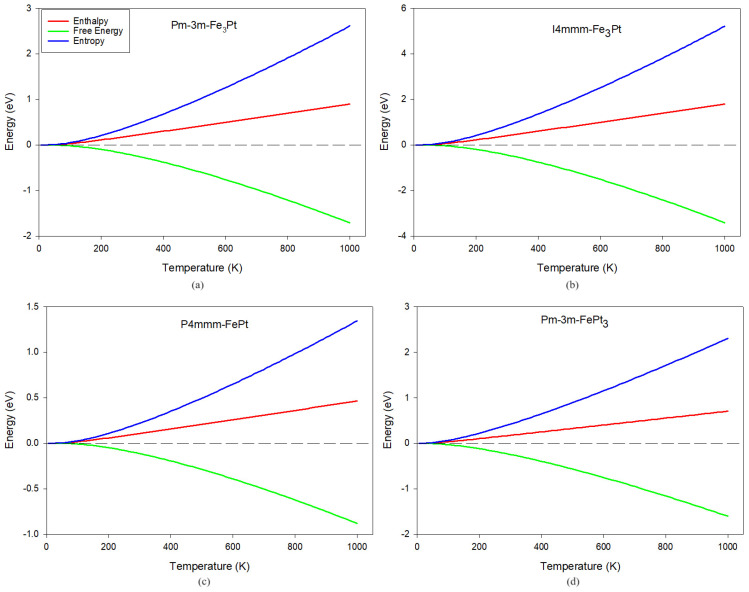
Thermodynamic properties of Fe-Pt alloys. (**a**) $Pm\bar{3}m$-Fe_3_Pt (**b**) I4/mmm-Fe_3_Pt (**c**) P4/mmm-FePt (**d**) $Pm\bar{3}m$-FePt_3_.

**Figure 4:**
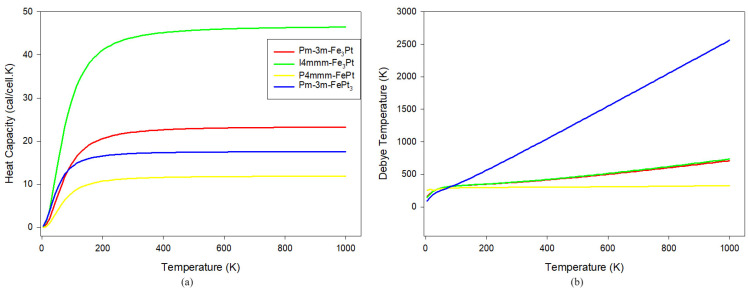
(**a**) Isochoric heat capacities and (**b**) Debye temperature vs. temperature for Fe-Pt alloys.

These three thermodynamic potentials as a function of temperature are plotted in Figure 3. From our graphical representations, we observed that entropy and enthalpy rise with temperature, while the free energy decreases. The I4/mmm-Fe_3_Pt alloy shows the largest entropy values, alluding to the high mobility of atoms within the lattice structure, while the P4/mmm-FePt has the lowest, suggesting a more compact structural arrangement and thermal stability and conductivity. This is consistent with the phonon dispersion spectra predictions where  P4/mmm-FePt showed dynamical stability. The free energies are negative and continue to decrease with temperature, implying thermodynamic stability [49], which corroborates the heats of formation in the previous communication [3]. Figure 4a shows the variation in isobaric heat capacity ($C_{v})$ as a function of temperature (0–1000 K). Heat capacities are critical in determining the thermal behavior of solid-state materials and are strongly dependent on intermolecular bonds. Materials with strong intermolecular bonds tend to have higher heat capacities as more energy is required to disrupt these bonds and increase the temperature. As the temperature increases, the heat capacities of Fe-Pt alloys show uneven characteristics. At temperatures below 200 K, $C_{v}$ increases exponentially and gradually scales off toward the Dulong–Petit boundary at higher temperatures. This behavior is common in most solid-state materials [19,21,52]. In the temperature range, I4/mmm-Fe_3_Pt (~46 cal/cell·K) shows the highest $C_{v}$ value, followed by $Fm\bar{3}m$-Fe_3_Pt (~23 cal/cell·K), $Fm\bar{3}m$-FePt_3_ (~17 cal/cell·K), and P4/mmm-FePt (~12 cal/cell·K), respectively. Interestingly, in temperatures below 80 K, the heat capacities are consistent with the bond length (see Table 1). An alloy with the shortest bond length (I4/mmm-Fe_3_Pt) exhibits the highest $C_{v}$ value, while an alloy with the longest bond length (P4/mmm-FePt) has the lowest value, further confirming a compact lattice structure on the latter.

The variation in the Debye temperature ($\theta_{D})$ as a function of temperature is shown in Figure 4b. Our results show a very strong linear increment relation between $\theta_{D}$ and T for $Pm\bar{3}m$-FePt_3_ and a moderate linear increase for I4/mmm-Fe_3_Pt and $Pm\bar{3}m$-Fe_3_Pt. Interestingly, P4/mmm-FePt shows a nearly constant $\theta_{D}$ vs. T relationship with a small standard deviation within 14, suggesting that the modes of vibration are compact and thermal stability is achieved for P4/mmm-FePt, which is in good agreement with the phonon dispersion spectra and entropy predictions. The Debye temperature decreases with Pt content, which is in good agreement with related DFT studies [19,53]. Moreover, $\theta_{D}$ of $Pm\bar{3}m$-FePt_3_ is highly affected by temperature, indicating the increase in atomic mobility and vibrations, which is consistent with the negative phonon modes of vibration.

## 4. Conclusions

The current study has successfully conducted ab initio simulations on the bimetallic Fe-Pt alloys to gain insights into the intrinsic factors underlying their mechanical, dynamical, and thermodynamic behavior, as well as to derive their stability trends. Moreover, we have established a link between the lattice dynamic and thermodynamic properties. All Fe-Pt alloys were predicted to be mechanically stable since they satisfied the Born stability conditions for cubic and tetragonal crystal lattices. Moreover, Fe-Pt alloys are characterized by the presence of sizeable anisotropy rising from the discrepancies in elastic constants and deviation of the universal anisotropy index from zero, which may give rise to a type of anisotropy called magnetocrystalline anisotropy. The prediction of mechanical stability and anisotropy is in good agreement with the thermodynamic stability and sizeable magnetocrystalline anisotropy energies reported in our previous communication. Computation of phonon dispersion spectra showed that the equiatomic L1_0_ P4/mmm-FePt is dynamically stable at ambient conditions, while the other compositions show soft modes along high-symmetry directions of the Brillouin zone. This study revealed that the negative vibrational modes predominantly emanate from Fe atoms in the highly dynamically unstable $Pm\bar{3}m$-FePt_3_ alloy, while dynamical instability is not pronounced in I4/mmm-Fe_3_Pt since the negative vibrations are not along the center of the Brillouin zone. Moreover, I4/mmm-Fe_3_Pt shows excellent mechanical stability, ductility, and thermal properties. Furthermore, the temperature versus entropy plots indicated that dynamically unstable alloys exhibit high atomic mobility as entropy exponentially increases with temperature, leading to the disordering of the lattice structure. This was corroborated by the heat capacity and Debye temperature plots. The free energies were predicted to be negative and to continue to decrease with temperature, implying thermodynamic stability in all the alloys. In addition to the current conclusions, it is of interest to perform ternary alloying of FePt with other transition metals such as Mn, Co, and Ni to determine any potential enhancement of the desired properties.

## Figures and Tables

**Table 1:** Calculated lattice constants, elastic constants, and moduli for the four ordered bimetallic Fe-Pt alloys. Available experimental data are provided in parentheses.

Parameter	$Pm\bar{3}m$-Fe_3_Pt	I4/mmm-Fe_3_Pt	P4/mmm-FePt	$Pm\bar{3}m$-FePt_3_
**Lattice Parameters (Å)**				
a (Å)	3.735 (3.72) [10]	5.276	2.725 (2.728)	3.914 (3.87) [22]
c (Å)	-	-	3.784 (3.85) [22]	-
**Bond Length (Å)** [4]				
Fe-Pt	2.641	2.638	2.700 (2.667)	2.768
Fe-Fe	2.641	2.638	-	-
Pt-Pt	-		-	2.768
**Elastic Constants (GPa)** [22]				
$C_{11}$	206.344 (238.8)	259.280	346.778 (304.2)	301.055 (325.8)
$C_{33}$		209.513	292.106 (242.0)	
$C_{44}$	85.957 (90.46)	68.757	113.855 (106.5)	105.108 (90.4)
$C_{66}$		35.522	48.38190 (40.8)	
$C_{12}$	170.625 (184.1)	90.790	73.457 (222.6)	183.094 (230.1)
$C_{13}$		152.871	161.090 (197.3)	
$C^{\prime }$	17.859	84.245	136.66	58.981
B	182.531	168.908	197.102	222.415
G	46.379	51.670	87.423	83.360
λ	151.612	134.461	138.820	166.842
E	128.274	140.667	228.488	222.306
σ	0.383	0.361	0.307	0.333
$A^{U}$	1.624	1.082	0.891	0.412
$\theta_{T}$	276.697	298.023	330.790	291.904
H	2.931	3.907	8.650	6.906
G/B	0.254	0.306	0.444	0.375

**Table 2:** Formulae for thermodynamic quantities.

$E\left(T\right)=E_{tot}+E_{zp}+\int \frac{\hbar \omega }{e^{\left(\frac{\hbar \omega }{kT}\right)}-1}F\left(\omega \right)d\omega ,$	Enthalpy
$F\left(T\right)=E_{tot}+E_{zp}+kT\int F\left(\omega \right)\left[1-e^{\left(-\frac{\hbar \omega }{kT}\right)}\right]d\omega$	Free energy
$S\left(T\right)=k\left\{\int \frac{\frac{\hbar \omega }{kT}}{e^{\left(\frac{\hbar \omega }{kT}\right)}-1}F\left(\omega \right)d\omega -\int F\left(\omega \right)\ln\left[1-e^{\left(\frac{\hbar \omega }{kT}\right)}b\right]d\omega \right\}$	Entropy
$C_{v}\left(T\right)=k\int \frac{{\left(\frac{\hbar \omega }{kT}\right)}^{2}e^{\left(\frac{\hbar \omega }{kT}\right)}}{{\left[e^{\left(\frac{\hbar \omega }{kT}\right)}-1\right]}^{2}}F\left(\omega \right)d\omega$	Heat capacity
$C_{\nu }^{D}=9N{k\left(\frac{T}{\theta_{D}}\right)}^{3}\int_{0}^{\theta_{D}/T}\frac{x^{4}e^{x}}{{\left(e^{x}-1\right)}^{2}}dx$	Debye temperature

where $E_{zp}$, k, ℏ, $F\left(\omega \right)$, $\theta_{D}$, and N are zero-point vibrational energy, Boltzmann constant, Planck constant, phonon density of states, Debye temperature, and number of atoms per cell, respectively.

## Data Availability

The generated data can be obtained from nlethole@ufh.ac.za or ndandulethole@gmail.com.
